# Framework for Modelling Economic Impacts of Invasive Species, Applied to Pine Wood Nematode in Europe

**DOI:** 10.1371/journal.pone.0045505

**Published:** 2012-09-20

**Authors:** Tarek Soliman, Monique C. M. Mourits, Wopke van der Werf, Geerten M. Hengeveld, Christelle Robinet, Alfons G. J. M. Oude Lansink

**Affiliations:** 1 Business Economics, Wageningen University, Wageningen, The Netherlands; 2 Alterra, Wageningen UR, Wageningen, The Netherlands; 3 INRA, UR 633 Zoologie Forestière, Orléans, France; 4 Crop and Weed Ecology Group, Centre for Crop Systems Analysis, Wageningen University, Wageningen, The Netherlands; Tel Aviv University, Israel

## Abstract

**Background:**

Economic impact assessment of invasive species requires integration of information on pest entry, establishment and spread, valuation of assets at risk and market consequences at large spatial scales. Here we develop such a framework and demonstrate its application to the pinewood nematode, *Bursaphelenchus xylophilus*, which threatens the European forestry industry. The effect of spatial resolution on the assessment result is analysed.

**Methodology/Principal Findings:**

Direct economic impacts resulting from wood loss are computed using partial budgeting at regional scale, while impacts on social welfare are computed by a partial equilibrium analysis of the round wood market at EU scale. Substantial impacts in terms of infested stock are expected in Portugal, Spain, Southern France, and North West Italy but not elsewhere in EU in the near future. The cumulative value of lost forestry stock over a period of 22 years (2008–2030), assuming no regulatory control measures, is estimated at €22 billion. The greatest yearly loss of stock is expected to occur in the period 2014–2019, with a peak of three billion euros in 2016, but stabilizing afterwards at 300–800 million euros/year. The reduction in social welfare follows the loss of stock with considerable delay because the yearly harvest from the forest is only 1.8%. The reduction in social welfare for the downstream round wood market is estimated at €218 million in 2030, whereby consumers incur a welfare loss of €357 million, while producers experience a €139 million increase, due to higher wood prices. The societal impact is expected to extend to well beyond the time horizon of the analysis, and long after the invasion has stopped.

**Conclusions/Significance:**

Pinewood nematode has large economic consequences for the conifer forestry industry in the EU. A change in spatial resolution affected the calculated directed losses by 24%, but did not critically affect conclusions.

## Introduction

A quantitative economic impact assessment of invasive species requires spatial integration of information on the potential for establishment, spread and impacts of the pest, which is a novel and challenging area in pest risk assessment [1]. Difficulties of integrating spread and economic impacts arise from unavailability of data on pest population densities, lack of knowledge on the relationship between those densities and expected yield reduction or quality loss, and difficulties in up-scaling the impacts from field to market level. Several studies have been devoted to the development of biological spread models [2]–[4] or economic evaluation models to estimate economic impacts (e.g. field and market scale) given predefined pest infestation rates [5]–[7]. Only few quantitative studies have integrated spread with impacts [8]–[10], and none of them accounted for the economic impacts at market level.

Wood production in forestry is vulnerable to invasive species, and enormous economic consequences have been reported. In the US, annual losses of forest products caused by invasive species exceed $4.2 billion [11] while in China these are estimated at $2.2 billion [12] and in Canada at $9.6 billion [13]. Invasive forest pests could lead to similar massive economic impacts on the European continent.

Pine wood nematode (*Bursaphelenchus xylophilus*) [14], [15] is recognized worldwide as a major forest pest [16]. Originating in the US, pine wood nematode (PWN) has spread to East Asia [17], Portugal [18], and North West Spain [19]. The nematode can reproduce quickly at high temperatures in summer. Huge populations of the nematode develop in infested trees, impeding water transport and causing the symptoms of pine wilt disease (PWD). Ultimately, PWD results in the death of the infested trees. PWN is vectored from diseased to healthy trees by bark beetles in the genus *Monochamus*. Pines (*Pinus* spp.) are favoured hosts but other genera of conifers (*Abies, Picea, Larix, Cedrus* and *Pseudotsuga*) are also attacked [16].

Since May 2008, Portugal has been classified as a demarcated area for PWN and subjected to emergency measures set out in Decision 2006/133/EC to prevent the further spread of PWN in the European Union (EU) [20]. Despite an intensive containment program (i.e. PROLUNG) [21], recent inspections carried out by the Food and Veterinary Office (FVO) of the European Commission indicated that the applied emergency measures have been insufficient [22]. Moreover, Sweden, Finland and Spain notified PWN findings in pallets imported from Portugal [22]. Intensification of the control measures may thus be required to eradicate the pest in Portugal and prevent further spread to the rest of the EU. However, the intensification of control measures must be economically justifiable as stated in the World Trade Organisation Agreement on the Application of Sanitary and Phytosanitary Measures (WTO-SPS Agreement) [23], [24]. It is therefore important to make an objective quantitative assessment of the expected economic consequences on European forest production and downstream markets that may result from a possible future spread of PWN from Portugal.

The objective of this paper is twofold. The first objective is problem oriented: to assess the expected economic consequences after 22 years of an uncontrolled PWN infestation in the EU by integrating information on climate, spread of PWN and value of forestry assets in Europe. The results give insight in the distribution of losses among geographical regions and show the impact on social welfare at pan-European level. The second objective is methodological. As little information is available on the effect of spatial resolution on economic impact assessments, this study compares the results of different spatial and economic techniques to arrive at an evidence-based quantification of economic impacts. The comparison clarifies how spatial resolution and economic assessment method affect the results and the associated requirements in terms of effort and data.

## Materials and Methods

### Conceptual Framework to Model Economic Impacts of Invasive Species

**Figure 1:**
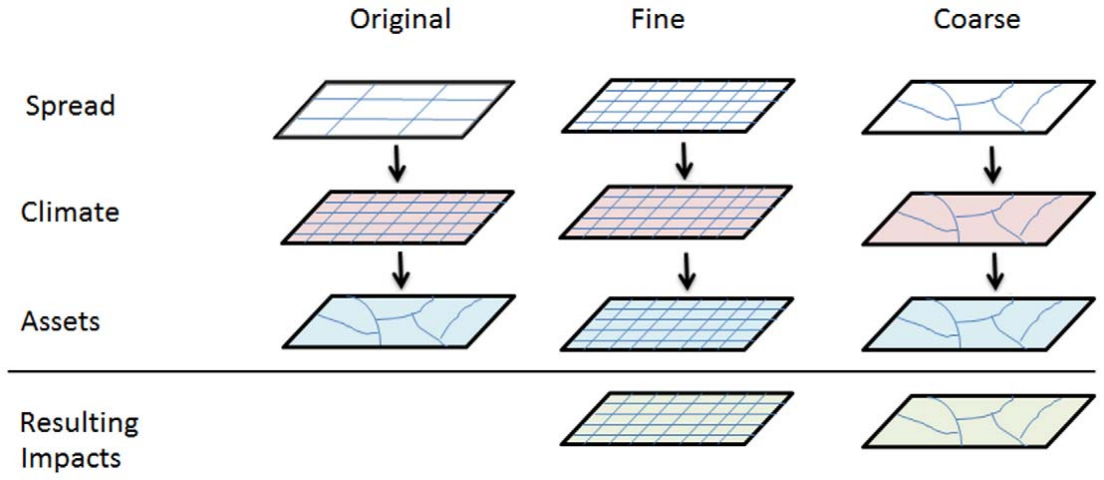
Conceptual framework for the risk assessment of invasive species. The framework consists of four modules, one for calculating the spatio-temporal spread of the pest, a second for climatic modelling to determine the areas suitable for impact (disease) expression, a third for modeling the spatial distribution and value of potential hosts or habitats and a fourth for calculation the resulting economic impacts.

The assessment of the economic impact resulting from a pest invasion in the EU requires an integration of information on pest entry, establishment and spread, valuation of assets at risk and market consequences at large spatial scales. The conceptual framework to model these economic impacts, therefore, consists of four modules, one for determination of the spatio-temporal spread of the pest, a second for climatic modeling to determine the areas where the climate is suitable for impact (disease) expression in infested hosts, a third for displaying the spatial distribution and value of potential hosts or habitats and a fourth for the calculation of economic impacts (Figure 1).

Data layers resulting from the spread, climate and host modules need to be integrated in a geographic information system (GIS) to enable the spatial quantification and mapping of the economic losses. Given the integrated information, module four calculates the expected economic consequences. These involve direct (i.e. host related) impacts such as yield reduction or quality loss within affected hosts, and additional production costs, as well as total, i.e. direct and indirect (non-host related) impacts such as changes in prices, demand and supply.

The scope of the application of the framework in this paper is to estimate the direct economic impact resulting from PWN affected trees in all coniferous host species present in the EU and the subsequent impact on the industrial round wood market resulting from the wood loss. Direct impacts are spatially indexed, and mapped, whereas the total impacts are calculated using a market model for the whole EU.

### Data Layers within PWN Framework

The first key data layer within the PWN framework describes the potential spread of PWN in Europe from the year 2008 till the year 2030. Information on spread was derived from the output of the process-based PWN spread model of Robinet et al. [4], [25], indicating the PWN spread potential from the initial infested sites in Portugal to the rest of Europe [4], [25]. The output of this model presents the presence or absence (yes or no) of PWN in individual grid-cells at the European level. Each grid-cell is 0.8° latitude×0.8° longitude, which covers on average 51 km^2^.

**Figure 2:**
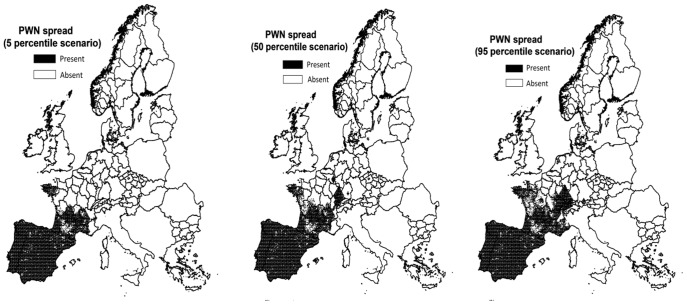
Simulated spread of PWN among Europe. Presented spread is based on the results of the 5^th^, 50^th^, and 95^th^ percentile replication of total extent of spread according to the spread model of Robinet et al. [25].

With the stochastic model of Robinet et al. [4], [25] two hundred replicate simulations were run to obtain a probability distribution of invaded area (per replication quantified as number of invaded cells) in the year 2030. The median invaded area covered 12,734 cells out of the total of 393,120 cells in Europe, while the 5^th^ and 95^th^ percentile invaded areas covered 11,445 and 14,448 cells, respectively (Figure 2). The median result was used in the assessment calculations. In the uncertainty analysis the 5^th^ and 95^th^ percentile were used to explore the sensitivity of economic impact to variation in spread.

The second data layer describes climate suitability. The key variable is temperature as the development of pine wilt diseases (PWD) is sensitive to summer temperatures [26]. Average summer temperature data (i.e. mean of July and August) over the years 1950–2000 were obtained from the WORLDCLIM database at 1 km^2^ resolution [27]. Data from PWN outbreaks in North America and Japan indicate that trees die due to PWD if temperatures are higher than 20°C for at least 8 weeks [28]–[30]. If PWN is present, but temperature is lower than the threshold for symptom expression, no PWD will be expressed.

**Figure 3:**
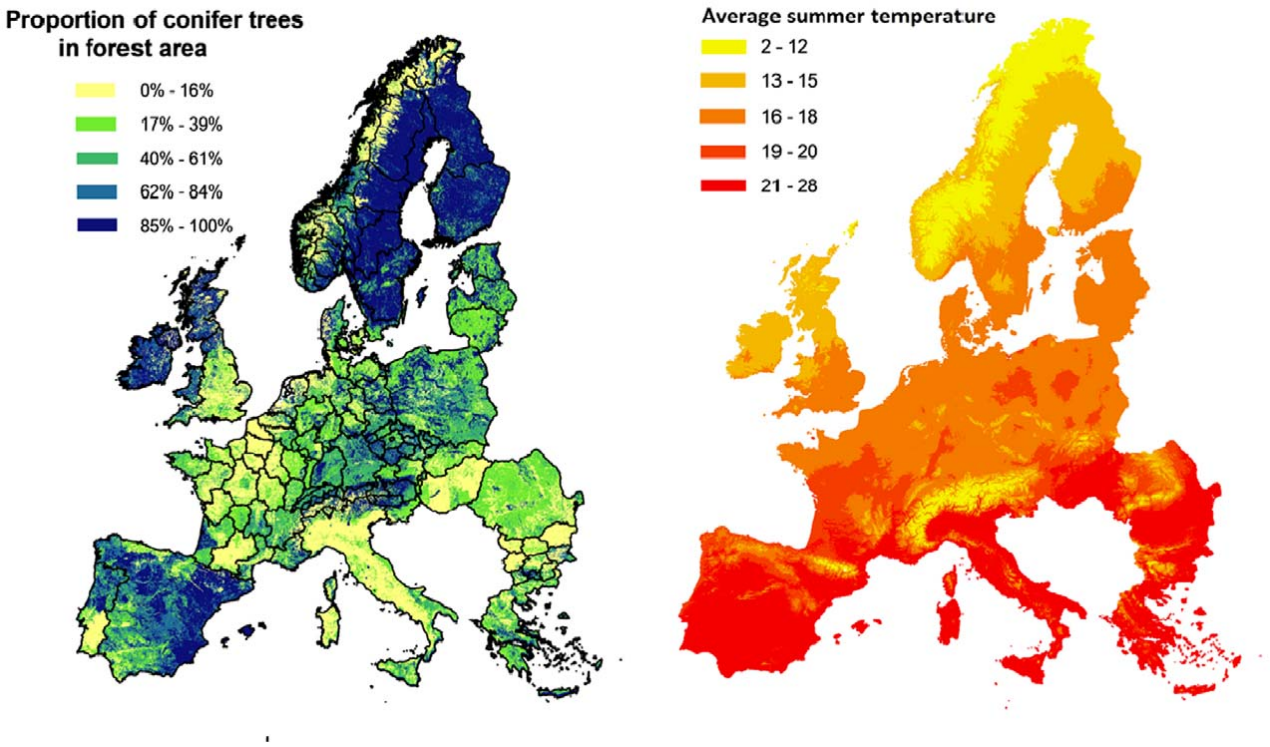
Proportion of conifer trees in forest at 1 km^2^ resolution (left) and average summer temperature in Degrees Centigrade (right).

The third data layer is the distribution of the assets (hosts) at risk within the EU. Host data were extracted from the European Forest Information Scenario Model - EFISCEN [31], [32], containing conifer forestry information for 25 countries of the EU plus Switzerland and Norway (Figure 3). Data representing the situation in Malta and Cyprus were not available and were therefore not included in the study. Data on Spain, Portugal and Italy were only available at country level, while data for the rest of Europe were represented at the more refined NUTS-1 or NUTS-2 region level (NUTS; Nomenclature of Territorial Units for Statistics) [33]. Only those conifer species that are susceptible to PWN infestation [16] were considered as assets at risk. The vulnerability of these assets and, therefore, the value at risk vary with the species related level of PWN susceptibility [16] and age [34], [35]. We classified the trees according to three levels of susceptibility (viz. susceptible, intermediate or resistant) and two classes of age (< = 20 years or >20 years), resulting in six vulnerability classes.

### Spatial Integration of Data Layers

The data of different layers were integrated at two levels of resolution, coarse and fine, to explore sensitivity of calculated impacts to spatial resolution adapted in the modelling. In the coarse resolution analysis, the units are NUTS-1 and NUTS-2 regions, depending on the level of detail at which the data are available from the original data source on host distribution. The coarse resolution analysis accounts for 117 NUTS regions. For the fine resolution analysis, the units are 1×1 km squares grid cells of which there are 3,856,062 in the modelled spatial domain. By the use of ArcGIS Desktop 9.3 information in the three data layers (spread, temperature and assets) were up- or downscaled as required to attain the desired level of resolution.

For the coarse resolution analysis, the three basic data layers were upscaled to the NUTS region level. The average summer temperature was calculated for NUTS regions by averaging across the grid-cells in each NUTS region. The presence or absence indicator of PWN was scaled up by calculating the proportion of infested 0.8×0.8 degree grid cells within each NUTS region. Assets at risk categorized by vulnerability class were already presented at the NUTS region level.

In the fine resolution analysis, data were integrated at a 1 km^2^ resolution. Temperature data were originally available at 1 km^2^ level but other variables needed to be downscaled. The value of the spread indicator in each 1 km^2^ grid (presence/absence) was obtained from the source cell of the spread model (0.8°×0.8°), assuming that presence in the source cell implied presence in each 1 km^2^ grid cell within it. Assets at risk were known by NUTS region. Damage at NUTS level was obtained by multiplying the value at risk in the region by the proportion of 1 km^2^ cells that met two criteria, 1) infested with PWN and 2) temperature higher than the threshold value for expressing PWD (see direct impacts). This total impact in a NUTS region was then distributed over the 1 km^2^ cells that met the two criteria for impact, assuming a homogenous distribution of the production value within the NUTS region.

### Economic Impact Module PWN Framework

A partial budget (PB) analysis was applied to calculate the direct impacts of a PWN infestation by assessing the expected loss in the standing stock available for round wood production in the EU resulting from 22 years of uncontrolled PWN spread. The direct economic impacts were estimated from 2008 up till year 2030 in line with the time horizon of the spread model. We consider only the value of lost wood and ignore the costs of mitigation which can – in principle – be included in PB analysis [36].

A partial equilibrium (PE) analysis was conducted to estimate the total impact or change in social welfare for the whole of the EU as a consequence of an affected wood supply destined for round wood production. PE analysis accounts for the phenomenon that changes in round wood production will trigger price changes that affect supply and demand. As a result of increased prices, some of the direct economic impacts may be passed on to consumers, which reduces consumer welfare but increases the welfare of producers.

While results of PB analysis are local and therefore spatially indexed, the results of PE analysis are aggregated values over the whole EU.

#### Direct economic impacts

The loss in standing stock available for round wood production was determined by the expected mortality rates as a consequence of the occurrence of PWD. In the valuation of the loss, it was assumed that trees expressing PWD are completely worthless, whereas healthy or symptomless trees retain their value.

Trees of 20 years or younger, and classified as ‘susceptible’, ‘intermediate’ or ‘resistant’ species were assigned default mortality rates of, respectively, 100% [26], 80% [34], [37] and 50% [38]. Trees older than 20 years in these same susceptibility classes were assigned slightly lower mortality rates of, respectively, 90% [39], [40], 70% [26], [20] and 40% [38].

The direct impact assessment assumes that PWN spreads after an invasion in 2008, without (1) any regulatory control measure in place, or (2) a change in the structure of the standing stock. The direct impact is expressed in terms of the total loss in production volume, accumulated over the time period of interest. For the year 2030, i.e. after 22 years of spread this is equal to:
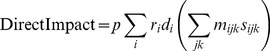
(1)where

*r_i_ = *proportion infestation with PWN after 22 years in polygon *i*

*d_i_* = indicator (0 or 1) for temperature above (1) or below (0) temperature threshold for expression of pine wilt disease (PWD)

*m_i_*_ = _mortality rates for trees of age class *j* and susceptibility class *k* in polygon *i*

*s_i_ = *standing stock available for wood production per age class *j* and susceptibility class *k* in polygon *i*.

p_ = _market price of round wood

The summation over age and susceptibility classes of trees is made for each polygon to calculate the overall value of assets at risk in a polygon. The proportion of infestation is calculated from the spread model output, while the indicator value for PWD expression is calculated from the temperature model for each polygon. In the fine resolution analysis, each polygon is a 1×1 km square, while in the coarse resolution analysis, each polygon is a NUTS region.

#### Total economic impact

**Figure 4:**
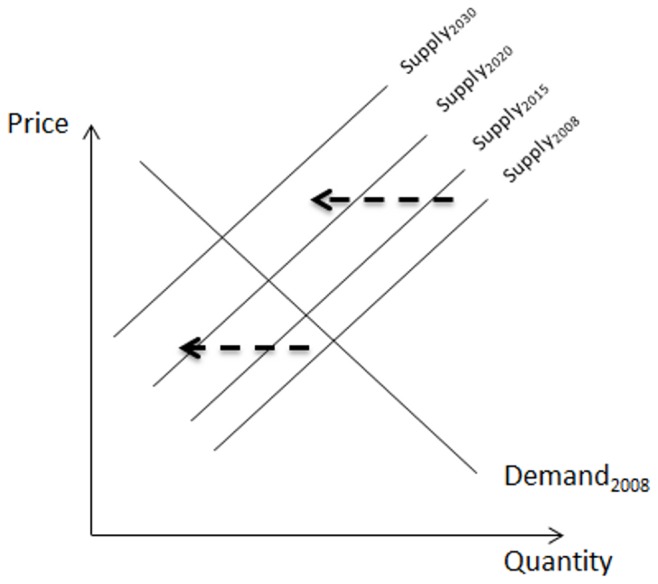
The partial equilibrium model. The annual shift in the supply side based on the accumulated loss in standing stock (2008–2030). The dashed arrow represents the direction of the vertical shift in the supply side of the market model.

Partial equilibrium (PE) modeling was used to assess the total (direct and indirect) impacts of PWN, viz. the impact on social welfare. PE is a powerful tool to evaluate the welfare effects on participants in a market which is affected by a shock like an introduction of a pest. The approach is based on defining functional relationships for supply and demand for the commodity of interest to determine the market equilibrium or, in other words, the combination of prices and quantities that maximizes social welfare [41] (Figure 4). A shock, like a pest invasion, may lead to a loss in yield and an increase in production costs, resulting in an upward shift in the supply curve as indicated by Figure 4. This shift in the supply curve alters the equilibrium point, implying a decrease in quantity supplied and an increase in market price. By measuring the differences in equilibrium price and quantity before and after the shock, the total impact of a shock is determined.

In the PWN assessment, total impacts were calculated for the whole EU, since there is one internal market for wood in the EU. The focus is on the conifer industrial round wood market which represents 79% of the total round wood market, the other 21% being for fuel wood and charcoal [42]. In the PE model, it is assumed that (1) conifer round wood in the EU and in the rest of the world (ROW) are perfect substitutes and their respective prices differ only due to transportation costs and tariffs and, (2) the EU market for conifer round wood is perfectly competitive, implying product homogeneity.

Within the PE model, the demand and supply in the EU of conifer industrial round wood are defined by the following equations 2a–2g (based on [43]). The first equation (2a) describes the demand (*D*) in the EU market as a function of EU price (*P*).
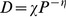
(2a)Where η is the price elasticity of demand and χ is scale parameter. The supply in the EU market has two components (equation 2b): supply by affected producers (*SA*) and supply by non-affected producers (*SN*).



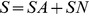
(2b)The supply by non-affected producers (*SN*) depends on the price *P*, with supply elasticity *θ* and scale parameter *β*, and is also determined by the proportion of producers that is not affected by the pest (1-z) (equation 2c):
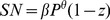
(2c)


The supply by affected producers (*SA*) depends furthermore on the proportional wood loss, *h*, caused by the disease, and by the reduced net price for the product that affected producers experience as a result of increased costs of production *ν* (e.g. for control or sanitation) (equation 2d):
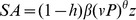
(2d)


Prices in the EU and the world market are linearly related where WP represents world price and *µ*, e.g. transport costs or tariffs (equation 2e):
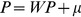
(2e)


The equilibrium condition for international trade (i.e. trade form EU to ROW) is expressed by two equations, 2f and 2g. The first of these (equation 2f) calculates export or import (*X*) as a difference between EU supply and demand.
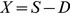
(2f)


The second equation (2g) expresses the relationship between international trade and world price, where *υ* is a scale parameter and *ω* is export/import elasticity.
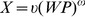
(2g)


Social welfare is calculated as the sum of monetary values of EU supply (producer) (eq.2b) and EU demand (consumer) (eq.2a). The change in social welfare due to PWN or total economic impact is determined by comparing the equilibrium results for a situation without PWN and a situation with PWN.

The expected annual total impact of PWN is calculated for the period 2008 till 2030, using data on prices and volumes in the round wood market from FAO statistics [44] and the expected proportion of infestation in time. The market price is the deflated EU market price of round wood of 2009, viz. 50.49 €/m^3^. Infestation levels at the EU level were obtained from the spread model. The shift in the market supply of round wood due to tree mortality was obtained from PB analysis (Figure 4). Currently, the average yearly tree removals for conifer industrial round wood production represent 1.8% of the forestry standing stock [42]. Based on the assumption that replacement of affected stock takes more than the evaluated 22 years before it will be effective for round wood production, it is assumed that the reduction in round wood supply in a year is equal to 1.8% of the accumulated loss in standing stock up to that year (as determined by equation 1). Inputs for the PE analysis are given in Table 1.

**Table 1:** Parameters on European industrial round wood production as used in the partial equilibrium model.

Parameter		Parameter	
Production (1000 m^3^) [44]	242,528	Consumption (1000 m^3^) [44]	249,101
Supply elasticity [54]	0.8	Demand elasticity [53]	−0.11
Producer price (€/m^3^) [44]	50.49	World price (€/m^3^) [44]	54.5
Excess supply (Import) elasticity	6.07		

### Uncertainty Analyses

Given the described module settings, the expected economic impacts were assessed with the direct economic impacts spatially indexed and mapped on a coarse (NUTS region) and fine resolution (1×1 km square), and the total economic impacts aggregated over the whole EU. The robustness of the estimated economic impact was evaluated by studying the extent to which the estimated direct economic impact at coarse resolution is affected by uncertainty in the spread, climate and host data layers. The following analyses were performed to account for this impact of data uncertainty;

- **Single parameter analyses.** A single parameter analysis was performed to study how the calculated direct economic impact is affected by (1) modelled variation in the spread of PWN, (2) variation in the literature with respect to the temperature threshold for PWD expression, (3) uncertainty as to the mortality rates for the tree hosts and (4) fluctuations in the market prices of industrial round wood. Sensitivity to variation in spread was assessed by comparing impacts at the median spread with impacts at the 5^th^ and 95^th^ percentile of spread (Figure 2, [25], [4]). Sensitivity to the temperature threshold for PWD expression was assessed by comparing impacts for three different thresholds values, viz.: 18°C, 19°C and 20°C [20], [45]. Sensitivity to mortality rates was assessed by constructing parameter sets representing low and high mortality as follows. For trees of 20 years or younger, minimum mortality rates for susceptible, intermediate and resistant trees were 60% [26], 60% [26] and 40% [38] respectively, and maximum rates 100% [26], 100% [25] and 50% [38]. For trees older than 20 years, minimum mortality rates for susceptible, intermediate and resistant trees were 50% [20], 50% [20] and 40% [38] respectively, and maximum rates 90% [20], 90% [39], [20] and 50% [38]. Impacts of market prices were evaluated by accounting for the lowest (50.49 €/m^3^) and highest (64.14 €/m^3^) deflated EU prices of industrial wood recorded in the period 2003–2009 [42].
- **Multi parameter analysis.** Worst and best cases were constructed by combining the parameter settings used in the single parameter analysis. The worst case assumes PWN spread based on the 95^th^ percentile spread value, an average summer temperature threshold of 18°C (i.e. low threshold), maximum mortality rate values and the highest market price for wood, while the best case assumes a PWN spread based on the 5^th^ percentile spread value, a temperature threshold of 20°C (i.e. high threshold), minimum mortality rate values and the lowest market price for wood.
- **Data layers analysis; removing temperature and spread constraints.** In order to assess the sensitivity of the results to availability of information on (1) temperature threshold and (2) introduction and spread of the nematode, direct economic impacts were recalculated assuming, firstly, that there is no temperature threshold required for PWD expression (PWD occurrence is only limited by dispersal) and secondly, that the point of entry of PWN invasion is not known (PWD occurrence is only limited by temperature). The first assumption is reflected empirically in the model by ignoring the temperature (climate) data layer and calculating impacts for all areas where PWN was present. The second assumption is reflected empirically by removing the spread data layer and calculating impacts for all areas in Europe which had an average summer temperature above 20°C.

## Results

### Assets at Risk

**Figure 5:**
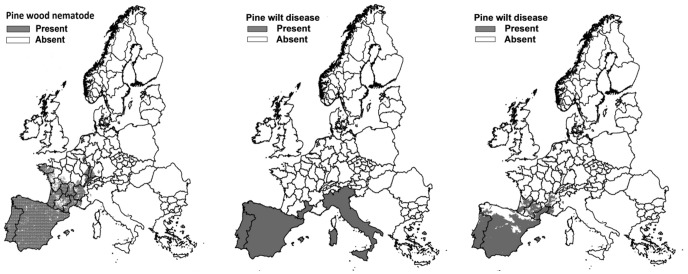
PWN and PWD potential spread cumulated over the period of 2008–2030. PWN potential spread at 0.8° latitude×0.8° longitude resolution (left), PWD potential spread at NUTS level, based upon summer temperature (middle), and PWD spread at 1 km^2^ resolution (right).

Integration of the spread, climate and host data layer defined the distribution of assets at risk. Susceptible conifer trees available for wood production represented 13,665 million m^3^ out of 24,594 million m^3^ of European forestry trees (Figure 3). Cells with presence of PWN and temperature above 20°C were cells that showed PWD. Depending on the resolution of the temperature data layer (NUTS and 1 km^2^), the PWD is expressed in 4 out of 117 NUTS regions in the coarse resolution and in 696,764 out of 3,856,062 (1 km^2^) cells in the fine resolution (Figure 5). These 696,764 cells were in 12 NUTS regions (Table 2).

**Table 2:** Infestation level and cumulative direct impact over 22 years of uncontrolled PWN spread in Europe.

Region	Coarse resolution		Fine resolution		
	Proportion infested area	Directimpact	Proportion infested area	Directimpact	Directimpact
	(%)	(M€/region)	(%)	(M€/region)	(€/km^2^)
Italy	0.15	30	0.15	30	43,136
Portugal	97.49	6,106	82.44	5,164	46,895
Spain	95.15	20,645	67.52	14,649	28,530
France					
Languedoc-Roussillon	84.65	1,084	50.32	644	28,831
Bourgogne	0	0	0.06	1	16,079
Poitou-Charentes	0	0	0.09	1	21,381
Aquitaine	0	0	19.86	1,219	90,611
Midi-Pyrenees	0	0	22.18	289	17,749
Limousin	0	0	1.71	15	30,124
Rhone-Alpes	0	0	14.71	215	19,626
Auvergne	0	0	0.19	3	31,792
Provence-Alpes Cote d’Azur	0	0	15.63	145	18,217
**Total (EU)**	**13.7**	**27,865**	**10.6**	**22,375**	

### Direct Impact

**Figure 6:**
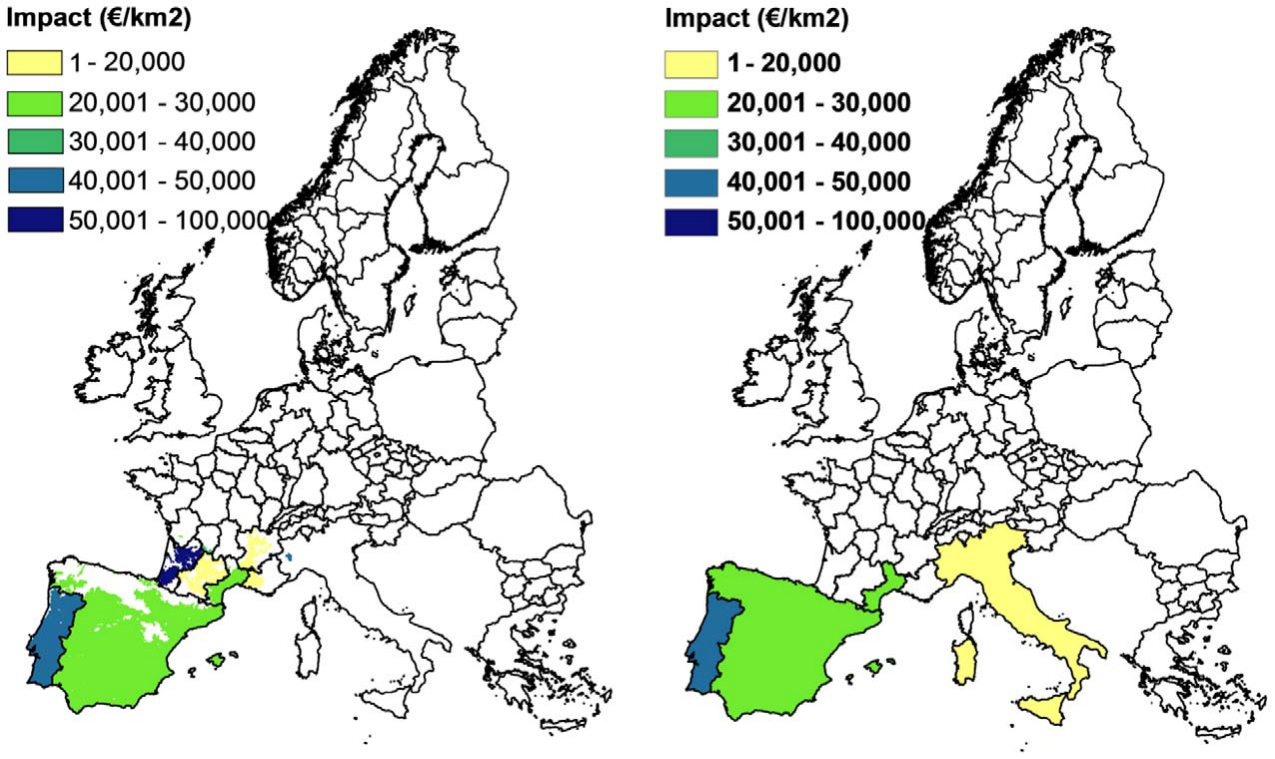
Cumulative direct economic impacts after 22 years of uncontrolled PWN spread in Europe. In the first panel, a fine resolution (1 km^2^) was used to conduct the analysis and to present the results. In the second panel, the analysis was conducted and presented at coarse resolution (NUTS level).

The PB analysis showed high expected timber losses in Portugal, Spain, Italy and France by 2030 (Table 2 and Figure 6). The cumulative wood loss in 2030 is estimated at €27 billion in the coarse resolution analysis at NUTS region level and at €22 billion in the fine resolution analysis at 1×1 km^2^ level, representing, respectively, 4% and 3.2% of the total value of PWN sensitive coniferous trees in the EU. Due to the width of the potential distribution of PWN and the availability of susceptible and intermediate host species in high densities, losses in standing volumes in Portugal and Spain are extremely high, respectively, 89% and 84% of total stock. In Italy, PWN is predicted to be present in only a few areas in the northwest part, thus reducing impact. Based on the coarse resolution analysis, only the southern part of France (Languedoc-Roussillon) is predicted to be affected by the nematode. The fine resolution analysis extends the impacted area in France to other southern regions (i.e. Bourgogne, Poitou-Charentes, Aquitaine, Midi-Pyrenees, Limousin, Rhone-Alpes, Provence-Alpes Cote d’Azur and the Auvergne). Of these regions, Aquitaine is predicted to have the highest impacts because of the presence of dense coniferous forests. Aquitaine is not predicted to be impacted in the coarse resolution analysis as the average summer temperature at the NUTS level was below the PWD temperature threshold of 20°C; however parts of Aquitaine have average summer temperature above 20°C, therefore the fine resolution analysis shows impacts in these parts of Aquitaine.

Overall, the cumulated direct impact estimated by the coarse resolution was 24% higher than estimated by the fine resolution. This is due to spatial aggregation resulting in a larger area where PWD can express. For example, Spain (as a NUTS region) has an average temperature above 20°C and the entire area of Spain was selected as endangered area in the coarse resolution analysis, while in the fine resolution analysis only 67% of the 1 km^2^ grid-cells of Spain was selected as endangered area.

**Figure 7:**
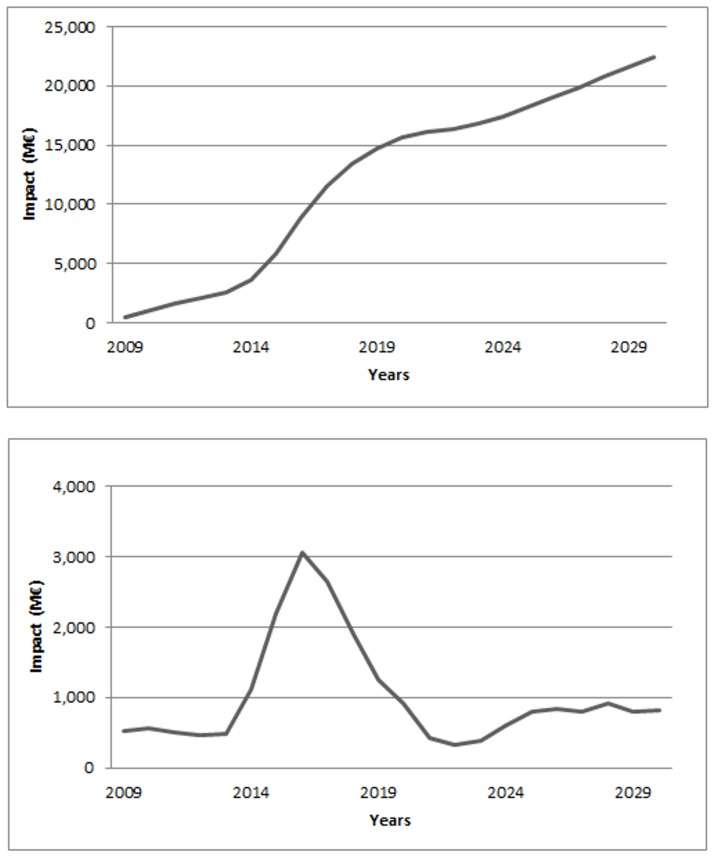
Direct impacts during 22 years (2008–2030) of uncontrolled spread of pinewood nematode in Europe. The first panel shows the cumulative direct impact, calculated as the total value of diseased trees over the entire infested area, i.e. the cumulative loss in harvestable stock. The second panel shows the marginal direct impact, i.e. the loss of harvestable stock due to the invasion process in each year.

Annual marginal analysis of the direct impact (i.e. based on the expected wood loss within a year) showed a sharp increase between the years 2014–2019, reaching its maximum in 2016 with a damage value of €3,068 million and a minimum in 2022 with a damage value of €329 million. At 2030 (i.e. the last year considered), the damage value was estimated at €816 million (Figure 7).

### Total Economic Impact

The results of the PE analysis showed that the reduction in domestic supply of industrial round wood (for both affected and non-affected producers) caused by an unregulated PWN invasion, led to an increase in the domestic market price, and a decrease in domestic demand. Annual net total welfare (impact on affected, non-affected producers and consumers) after 22 years of spread (i.e. a shift in the supply side of the market in 2030 due to the accumulated direct loss of 22 years) is reduced by € 218 million. The shortage in domestic supply (the gap between supply and demand) is covered by an increase in the imports and/or decrease in exports (i.e. change in net trade) (Table 3) and the increase in domestic price and changes in trade triggered an increase in the world price for round wood. The increase in prices caused an increase in supply leading to a new equilibrium in the industrial round wood market. Consumers suffered a reduction in surplus of € 357 million due to higher prices. Non-affected producers experienced a positive net impact as they benefited from the higher market price in the new equilibrium situation without suffering wood loss. Affected producers experienced a loss as the price increase did not wholly compensate for the reduction in production volume. On the whole, total producer surplus increased with €139 million. Coarse resolution analysis gave a 69% greater effect on annual total welfare than fine resolution analysis (Table 3).

**Table 3:** Annual total impacts due to pest invasion estimated by partial equilibrium modelling, based on direct impact assessment at coarse or fine resolution.

	Coarse resolution		Fine resolution	
	Absolute	%	Absolute	%
Supply (M m3)	−3.24	−1.3	−1.89	−0.8
Demand (M m3)	−1.27	−0.5	−0.77	−0.3
Price (€)	2.40	4.5	1.44	2.8
Net trade (M m^3^)	−2	−23.0	−1	−14.6
Consumer surplus (M €)	−597	−4.1	−357	−2.5
Producer surplus (M €)	228	3.2	139	2.0
Total welfare (M€)	−369	−1.7	−218	−1.0

**Figure 8:**
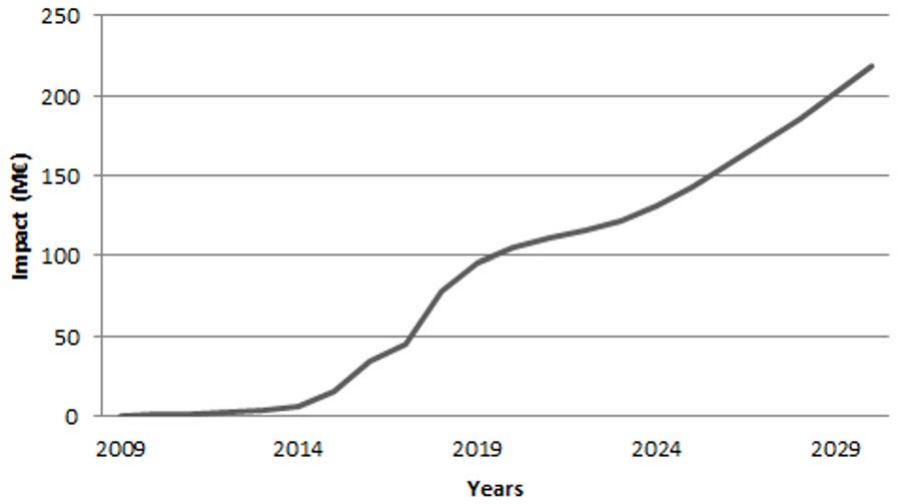
Annual total impact (i.e. net welfare) from 2008–2030 due to 22 years of uncontrolled spread of pinewood nematode in Europe, resulting from the cumulative reduction in harvestable stock.

Analysis of the total impact per year based on an accumulated shift in the supply side of the market, showed a net welfare reduction of 5 M€ in 2009, 142 M€ in 2019 and 218 M€ in 2030 (Figure 8).

### Uncertainty Analyses

i **Single parameter analysis.** The calculated direct economic impact as calculated at coarse resolution was insensitive to uncertainties in the potential spread of PWN but sensitive to the assumed temperature threshold for PWD expression, the tree mortality rates and market prices (Table 4).

**Table 4:** Parameter settings and results of the coarse resolution uncertainty analysis.

Parameters	Settings			Direct impact (M €)		
Spread (percentile)	5^th^	50^th^ *	95^th^	27,842	27,865	28,342
Temperature (°C)	18	19	20 *	35,020	34,353	27,865
Mortality rate (%)	minimum(40–60)	most likely *(50–100)	maximum(50–100)	16,264	27,865	28,636
Market price (€)	minimum *(50.5)		maximum(67.7)	27,865		37,363

* default setting.

The total economic impacts based on the 5^th^, 50^th^ and 95^th^ percentile spread settings were similar at approximately € 27 billion. Due to the modelling condition of having the point of entry in Portugal and the presence of high host densities in Portugal and Spain, variation in spread within southern Europe turned out to be minimal. Spread variation among the northern European regions was larger (Figure 2). However, due to the temperature threshold for PWD expression of 20°C, spread variation only resulted in a small change in PWD occurrence and therefore in the economic impact. Impacts were sensitive to the settings of the temperature thresholds as the highly impacted regions (Portugal, Spain and Aquitaine (France)) occur between the 20°C climatic zone (i.e. Portugal and Spain), and the 19°C climatic zone (i.e. Aquitaine). The difference in impacts between the 18°C and 19°C climatic zones was minimal due to the presence of few susceptible trees in areas that are (1) in the invaded area till 2030, and (2) have summer temperatures between 18 and 19°C. Therefore the increase in temperature threshold from 18 to 19°C resulted only in a small number of additional trees at risk (Table 4).

Impacts were also sensitive to mortality rates as any change in this parameter immediately affects the results. Any change in the market prices changed the direct impacts proportionally.

ii **Multi parameter uncertainty analysis.** The direct economic impact entailed a cumulative loss of € 35.9 billion in the worst case, and a cumulative loss of € 16.2 billion in the best case (Table 5). The level of uncertainty in each region is reflected by the difference in impacts between the best and worst case in comparison to the total value of trees within the corresponding NUTS region (Table 5). The results of this ratio suggested a high level of uncertainty in Aquitaine.

**Table 5:** Estimated direct impacts in the best and worst case scenario, given per NUTS region, in absolute as well as relative terms, as compared to the total value of trees within each region.

NUTS-2 region	Worst case		Best case		Difference
	(M€)	(%)	(M€)	(%)	(%)
Centre	124	7	0	0	7
Bourgogne	136	8	0	0	8
Pays de Loire	246	17	0	0	17
Poitou-Charentes	325	28	0	0	28
Aquitaine	5,290	80	0	0	80
Midi-Pyrenees	995	42	0	0	42
Languedoc-Roussillon	1,208	54	827	37	17
Provence-Alpes Cote d’Azur	210	14	0	0	14
Italy	33	0	20	0	0
Portugal	6,205	91	3,548	52	39
Spain	21,191	87	11,870	48	38
**Total**	**35,962**	**46**	**16,264**	**21**	**25**

iii **Data layers analysis; removing temperature and spread constraints.** Assuming that all infested trees will express PWD regardless of the location temperature, the estimated value of wood loss of susceptible trees equalled M€ 56.5 billion, while the value of wood loss of all susceptible trees available in EU areas with average summer temperature above 20°C, regardless of the locations invaded by PWN, represented a value of € 43.3 billion.

## Discussion

The results of this economic assessment demonstrate that an uncontrolled PWN invasion will lead to large economic consequences for the conifer forestry industry in the EU. From the current point of entry in Portugal an invasion is expected to affect 10.6% of the studied EU area by 2030. The cumulated wood loss after 22 years of unregulated spread, calculated in a fine resolution analysis, is estimated at €22 billion, representing 3.2% of the total value of PWN susceptible coniferous trees in the EU. The reduction in social welfare in 2030 is estimated at €218 million.

There is a large difference in the magnitude of the estimated direct and total impacts. This is because the direct impacts represent the reduction in value of standing forestry stock, while the total impacts refer to the changes in the yearly flow of wood to the round wood market. These flows represent 1.8% on average of the standing stock [42]. Therefore, losses in flow accumulate slowly and for a long period of time after the standing stock is considered as lost because of PWD. We do not consider in this study the recovery of rest value by planting resistant trees, because these would not be harvested within the time frame considered in this study (22 years). When the spread of PWN stops, the annual direct loss will disappear while the total impact will continue to increase until mitigation efforts become effective (i.e. replanted (resistant) trees flow to the round wood market).

The fine resolution analysis provided more plausible results in terms of size and distribution of the impacts, while requiring limited extra effort. The coarse resolution analysis did not identify Southern France as an area at risk as a result of averaging temperature over a large region, and therefore, it misrepresents the distribution of the impacts. Direct impacts estimated at a coarse resolution level were 24% higher than those obtained at a fine resolution level. The presented differences follow directly from the applied aggregation procedure on the temperature data layer.

While the difference in total impact as estimated by the coarse and fine resolution analyses did not alter the assessment of the riskiness of the pest, the difference in the geographical distribution of the impacts predicted by the two resolutions is quite critical and management relevant. The fine resolution analysis is more relevant to EU risk managers as it provides a more plausible assessment of the expected distribution of direct impacts within the EU.

Economic impacts were on the basis of (1) the predictions of the spread model for the presence/absence of the PWN and (2) a temperature threshold of 20°C for PWD. The sensitivity of the results to these two data layers was assessed in the data layers uncertainty analysis. The spread model was calibrated on the invasion history in China and should be refined in the future to determine more precise the potential expansion in Europe [4], [25]. Including no information on the potential spread implies that PWD infests all areas where host trees are available and the climate is suitable for expressing symptoms. This situation could arise if point(s) of entry other than Portugal are found. Absence of a temperature threshold means that PWD is assumed to infest all areas invaded by PWN, regardless of their summer temperature. With global warming (+1.8 to +4°C predicted between 1980–1999 and 2090–2099 [46]), the temperature constraint will be less restrictive in the future. When removing spread and temperature conditions, the cumulative direct economic impacts are massive: € 56.5 billion when the point of entry is unknown and €43.3 billion when there is no temperature restriction. Compared to the default impact of €27.8 billion, the availability of information on spread and climate as such did not critically influence the assessment of the risk posed by PWN. Nevertheless, the use of information on spread and climate demonstrated the geographical distribution of the impacts within the EU which is of value to EU risk managers. For instance, it allows a risk manager to compare the effectiveness of alternative management plans in terms of its temporal and spatial dimensions (e.g. early and late containment programs).

The economic impacts were estimated till the year 2030 conform the time horizon of the spread model. The time horizon of 22 years was considered long enough to allow the pest to show its invasion potential and short enough to have a reasonable technical processing time and an acceptable uncertainty level in the results [4].

Integrating spread and impacts is a challenging area in pest risk assessment [1]. The challenge arises from the fact that to quantify economic impacts, we need to know the areas of significant economic loss. Significant losses occur when the pest population densities exceed the economic injury level (EIL). The EIL is the population density at which the cost to control the pest equals the amount of damage it is likely to cause [47]–[49]. To integrate spread and impact, knowledge is required on: (1) the areas where pest population densities exceed the EIL and (2) the relationship between pest population densities and the likely level of yield or quality loss. A number of recent studies followed this approach [50], [51] as they used pest population densities generated by a spread model and link it with yield loss. In the absence of spread models which can be used to predict pest population densities spatially, climate data (or other agro-ecological information such as soil type or irrigation) can be used as a proxy [25]. As climate influences the growth rate of the pest population, climate data can be used to indicate pest density levels. However, climate cannot be used to reflect the change in the pest population density over time.

Extending the economic assessment to market models with open economies (e.g. partial equilibrium) rather than just stick at the field/producer level models (e.g. partial budgeting) is essential as the market power of large areas like the EU in the world trade and the importance of the resulting spill over effects cannot be ignored. However, the main obstacle is that partial equilibrium models and invasion ecology models present different spatial and dynamic scales [52] in addition to the unknown producers supply responses which play a critical role when scaling up impacts from field to market level.

The approach proposed in this study extends the risk mapping process from the establishment and spread phase to the economic impacts phase. Accordingly, our approach can be followed in pest risk analyses where there is a need to represent impacts not only in relative pest risk levels but also in terms of euros, in order to increase the transparency and objectivity of evaluated plant health measures as required by the international agreements on plant health and trade [23], [24].
